# Key predictors of COVID-19 vaccine hesitancy in Malaysia: An integrated framework

**DOI:** 10.1371/journal.pone.0268926

**Published:** 2022-05-23

**Authors:** Jason Wei Jian Ng, Santha Vaithilingam, Mahendhiran Nair, Li-Ann Hwang, Kamarul Imran Musa

**Affiliations:** 1 Department of Econometrics and Business Statistics, School of Business, Monash University Malaysia, Selangor, Malaysia; 2 Institute of Global Strategy and Competitiveness and Sunway University Business School, Sunway University, Selangor, Malaysia; 3 School of Medical Sciences, University Sains Malaysia, Kelantan, Malaysia; University of Haifa, ISRAEL

## Abstract

**Background:**

As the vaccination drive against the coronavirus disease (COVID-19) in Malaysia progresses rapidly, the main challenge will gradually shift from procuring and distributing vaccines to ensuring the broadest possible acceptance among all population segments. Therefore, this study used the integrated framework of the health belief model (HBM) and the theory of reasoned action (TRA) to investigate the predictors of intention to receive COVID-19 vaccines in Malaysia.

**Methodology:**

A market research company in Malaysia was engaged to collect data during June 11–20, 2021 using self-administered questionnaires via its online panel, ensuring a nationwide random sample of 804 respondents. A logistic regression was subsequently estimated to determine the significant predictors of vaccination intention.

**Results:**

The predictors that significantly affect COVID-19 vaccine hesitancy in Malaysia are age, susceptibility, religious beliefs, attitude, subjective norms, and trust in the vaccine. In particular, those who are more inclined to get vaccinated are older individuals, have a higher perceived risk of infection and social pressure to get vaccinated, have a positive attitude, and have high levels of trust in the vaccine. Individuals’ who perceive that their religious beliefs are against vaccination are more likely to exhibit uncertainty toward it.

**Conclusion:**

This study showed that although a large proportion of respondents indicated that they were willing to be vaccinated against COVID-19, there are concerns about religious barriers and trust in the vaccine that the relevant stakeholders should address. Campaigns should also focus on shaping the nation’s attitude toward COVID-19 vaccines, in parallel with encouraging people to use their social influence in helping those in their close circle who are unsure of vaccination to cross the line. These measures will prove to be pertinent as the nation begins to administer booster vaccines to tackle the waning effects of COVID-19 vaccines.

## Introduction

The World Health Organization declared the outbreak of the coronavirus disease (COVID-19) caused by the severe acute respiratory syndrome coronavirus 2 (SARS-CoV-2) as a global pandemic on March 11, 2020. Since then, as of November 2, 2021, the number of reported infections and deaths worldwide has reached a staggering 246 million and 5 million, respectively [1].

In the initial response to the pandemic, many countries adopted a “Covid-Zero” strategy of mandating strict lockdowns nationwide to curtail people’s movement in a bid to break the chain of transmissions. However, with the resurgence of cases due to the “Delta variant” of the virus, countries have shown signs of moving away from this strategy. They focus instead on increasing vaccine uptake to reach herd immunity, that is, a sufficient proportion of the population acquiring immunity [2–4].

As of November 2, 2021, Malaysia has recorded more than 2.4 million reported COVID-19 infections. As a reflection of the country’s purported inability to contain the pandemic, Malaysia was subsequently ranked 50th out of 53 economies in the 2021 Bloomberg Covid Resilience Ranking [5]. Nevertheless, despite a slow start to its vaccination rollout, Malaysia has emerged to have one of the highest vaccination rates globally, surpassing that of Singapore, Japan, South Korea, the United Kingdom, and the United States [6]. However, despite the increasing vaccination rate in the country, pockets of people are either unsure about vaccination or are unlikely to get vaccinated [7]. Therefore, as the vaccination drive against COVID-19 in Malaysia progresses rapidly, the main challenge will gradually shift from procuring and distributing vaccines to ensuring the broadest possible acceptance among all population segments.

Against this backdrop, in this study, our main objective is to investigate predictors of intention to receive the COVID-19 vaccine from a developing country’s (i.e., Malaysia) perspective, in which COVID-19 and vaccine hesitancy have adversely affected several population segments. To this end, to examine vaccine hesitancy we integrated the *health belief model* (HBM) and the *theory of reasoned action* (TRA) [8] frameworks, both of which have been independently employed to predict vaccination intentions [9–12]. The World Health Organization has defined vaccine hesitancy as “a delay in acceptance or refusal of vaccination despite vaccine availability” [13,14].

Notably, the HBM, developed in the 1950s, remains one of the most widely used frameworks to predict health behavior [15]. In the context of COVID-19, the HBM posits that the likelihood of individuals receiving a vaccine is determined by their perceptions regarding the severity of, and susceptibility to, the virus, and regarding the barriers to, and benefits of, vaccination. In contrast, the TRA postulates that an individual’s intention to get vaccinated is influenced by attitude and subjective norms [16]. In the current study, we relied on a nationwide sample of Malaysian adults to identify the HBM and TRA constructs associated with vaccine intention. Given that vaccine attitudes are also influenced by demographic factors [17], we also examine which demographic factors predict vaccine intention in Malaysia.

Earlier studies have assessed the acceptance of the COVID-19 vaccine in Malaysia [10,18,19]. However, this study differs from them in the following ways. First, contrary to the social media snowball sampling methods commonly adopted in prior research, this study leveraged the partnership of a market research company to collect data through a nationwide survey via random sampling. Second, the survey was conducted in early June 2021, shortly after Malaysia’s daily COVID-19 infections hit a new high of more than 8,000 [20], reflecting the worsening situation. By this time, Malaysia was also into the fourth month of its national vaccine rollout [21], and the COVID-19 vaccine was more readily available. These developments allow for a better assessment of people’s behavioral intention to get vaccinated. In contrast, earlier Malaysian studies were conducted when vaccines were either unavailable [10] or when the COVID-19 infection was not as severe as when this study was conducted [18,19]. Third, our multivariate model specification is derived from the integrated theoretical frameworks of the HBM and TRA, whereas prior studies employ primarily the HBM only [10,18]. We further augment our model by including other contextual factors, such as trust [22], vaccine preference [23], and the issuance of a vaccine passport to facilitate international travel [24], all of which may influence vaccine intention in Malaysia.

Malaysia has established a four-phased approach in its *National Recovery Plan*. Ultimately, the success of Malaysia’s roadmap to safely exit the pandemic and reopen its economy will hinge on the nation’s ability to reach high vaccination levels, which will facilitate coexistence with the virus in the endemic phase [25]. However, because vaccination against COVID-19 is not yet a mandatory requirement for residents, appropriate policies and strategies will need to be developed to attain widespread vaccine uptake. This challenge will become particularly acute as the vaccination rate reaches a saturation point. To this end, it is essential to understand the factors that encourage or hinder Malaysians from being vaccinated against COVID-19. The learnings from this study will also be useful to address future health pandemics.

## Methodology

### Data collection

Data were collected during June 11–20, 2021 from 804 Malaysian adults (81.8% response rate) aged 18 years and above. A marketing consulting firm in Malaysia was engaged to collect data using self-administered questionnaires via an online panel. Since Malaysia was under a nationwide lockdown during the time of collection, the consulting firm could not conduct fieldwork to collect data from the rural population, which is less connected to the internet. Therefore, the sample is skewed toward the more urban, connected population.

Similarly to Mohamed et al.’s [18] study in the Malaysian context, we used the Rasoft sample size calculator to determine the study’s sample size. A minimum of 385 participants were required at a 5% margin of error, 95% confidence level, and a 50% response distribution for an adult population of 23.5 million aged 18 and above.

The questionnaire was prepared in English and translated into Malay and Chinese by professional translators. The Malay and Chinese questionnaires were then back-translated to ensure that the original and translated questionnaires were similar in terms of content, clarity, and meaning [26]. The back-translation was compared with the original questionnaire to ensure reliability and validity. We also computed the content validity index (CVI) for the constructs. A panel of six experts evaluated the items for each construct on a scale ranging from 1 to 4 (1 = not relevant, 2 = not important, 3 = relevant, and 4 = important). Those who rated the items as either relevant or important are taken to agree to have the item in the construct. For all the constructs, except for intention to vaccinate, the content validity of the individual item (I-CVI) values is 1 and the mean CVI is 1. For the intention to vaccinate construct, only one item has an I-CVI of 0.83, and the overall mean CVI is 0.96. The mean CVI for each construct is within the criteria recommended by Lynn [27], that is, a minimum I-CVI of 0.78 for 6–10 experts and an overall mean CVI of 0.90 or higher.

### Measures

We developed the questionnaire using validated instruments from prior studies. We included a few self-developed items to capture perceptions specific to the Malaysian context. Our primary measure of interest was participants’ intention to get the COVID-19 vaccine. Drawing upon the HBM, we were interested in their perceptions of susceptibility and severity toward COVID-19. In the context of COVID-19, perceived susceptibility refers to individuals’ assessment of the risk of being infected, whereas perceived severity refers to their beliefs about the seriousness of infections [15]. Items measuring perceived severity were obtained from Chu and Liu [28] and Coe et al. [9], whereas measures of perceived susceptibility were adapted from Chu and Liu [28] and Yang [29].

Participants were also asked about their perception of the barriers to, and benefits of, getting vaccinated against COVID-19. This study defines perceived barriers as individuals’ assessment of the influences that hinder or discourage vaccination [15]. Perceived barriers were operationalized into four barriers: clinical, access, registration, and religion. The items measuring clinical and access barriers were adapted from Chu and Liu [28], Coe et al. [9], and Yang [29]. In contrast, the items used to measure registration and religious barriers were self-developed to cater to the Malaysian context. Perceived benefits are associated with the positive outcomes of vaccination [15] and were operationalized to comprise individual and community benefits. Instruments to measure perceived benefits were obtained from Chu and Liu [28].

We also included constructs such as attitude and subjective norms from the TRA. In the context of this study, attitude refers to an individual’s evaluative effect about getting the COVID-19 vaccine [30]. Measures of attitude were adapted from prior studies that have also applied them in the context of vaccination intention [28,29]. Subjective norms refer to individuals’ perception that most people who are important to them think they should or should not get vaccinated against COVID-19 [16]. To measure subjective norms, we referred to the items used by Chu and Liu [28] and Venkatesh et al. [31]. Last, we considered other variables of interest, including trust, vaccine preference, and vaccine passport. Measures of trust include trust in the vaccine process [32] and in information sources [33]. Questions used to measure vaccine preference and vaccine passport were self-developed. In particular, for vaccine preference, respondents were asked which COVID-19 vaccine they would prefer to receive. For vaccine passports, respondents were asked whether they would be more likely to get vaccinated if they were to subsequently receive a vaccine passport for international travel.

The questionnaire was divided into two sections. Section A comprised items measuring vaccination intention, perceived severity, perceived susceptibility, perceived barriers, perceived benefits, attitude, subjective norms, and trust. These items were measured on a 5-point Likert scale. All items employed in the questionnaire were modified to suit the study context. Section B consisted of questions regarding the demographic characteristics of the respondents (see S1 Questionnaire in S1 Appendix for the full list of items).

### Data analysis

Since the number of respondents who are unlikely to be vaccinated is small (n = 15; 1.86%), this study’s dependent variable regarding respondents’ intention to be vaccinated could be categorized into two possible outcomes: “Unlikely/Unsure” and “Likely.” Therefore, we estimated a logistic regression model to examine differences between respondents who were unlikely and unsure to be vaccinated, and those who reported intention to be vaccinated.

Except for gender, education, income, vaccine preference, and vaccine passport, all other predictors were treated as continuous variables. Regarding the HBM and TRA constructs measured using multiple indicators, we combined the 5-point Likert scale responses of the individual items to create a composite measure by taking the mean. Gender had two levels of identification used for the analysis (male, female), with females as the reference category. Regarding education, respondents were grouped according to their highest level of educational attainment, given by secondary or lower education (reference category), diploma education, or tertiary education. Income was measured using five classes: “Below RM1,000” (reference category), “RM1,000–RM3,999,” RM4,000–RM6,999,” “RM7,000–RM9,999,” and “Above RM10,000.” For vaccine preference, respondents were invited to indicate whether they preferred AstraZeneca (reference category), Moderna, Pfizer, Sinovac, or any vaccine. Last, respondents indicated whether they would be more likely to be vaccinated if a vaccine passport were issued by responding “Yes,” Unsure,” or “No” (reference group).

Data analyses were conducted using R version 4.0.3 and *p*-values less than 0.05 were considered statistically significant.

### Ethics statement

Ethics approval was obtained from the Monash University Human Ethics Committee (Project ID: 28249). We obtained written consent from participants before administering the questionnaire. Details of each participant (i.e., name and other personal identifiers) were de-identified.

## Results

**Fig 1:**
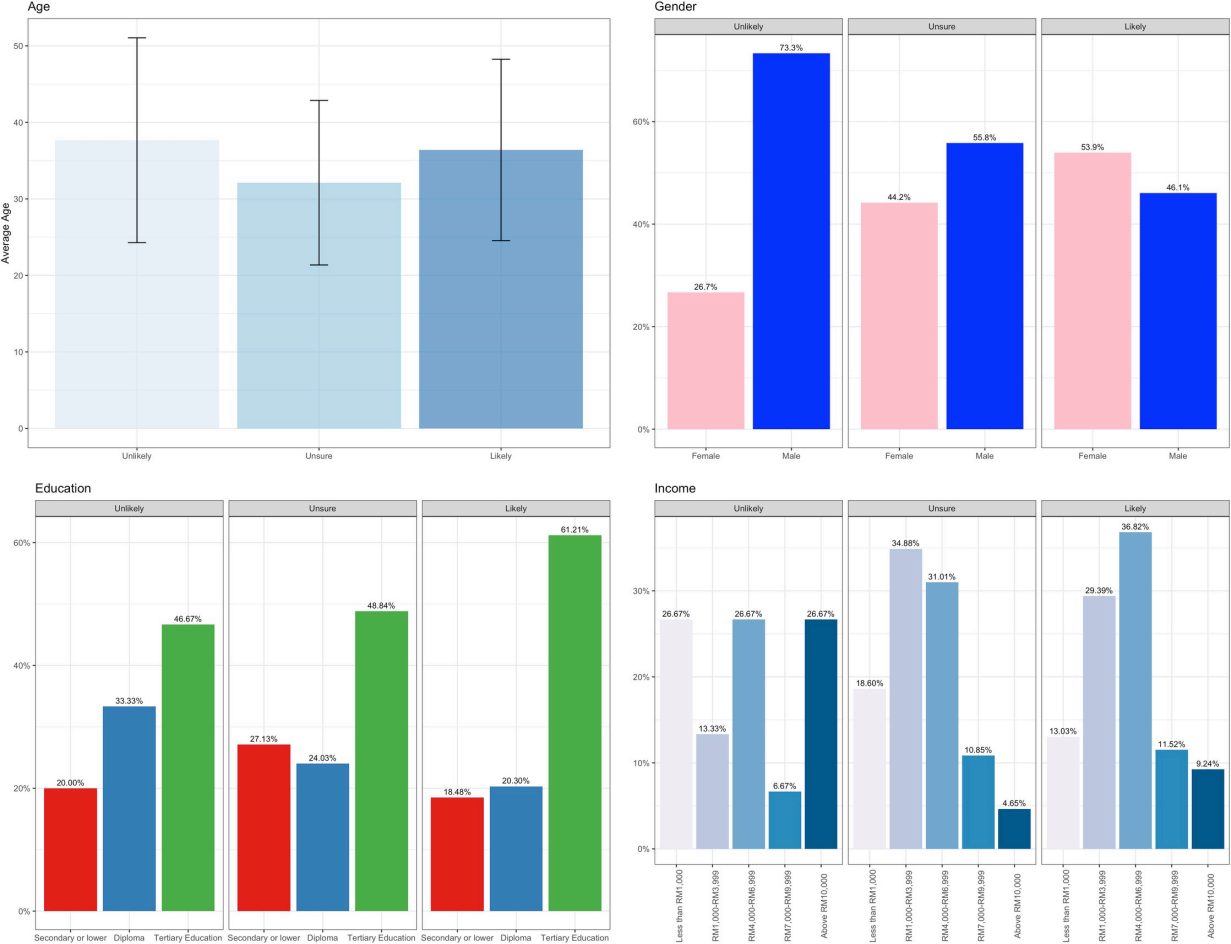
Demographic of respondents according to vaccination intention.

Data from a total of 804 participants were analyzed. In Fig 1 and Table 1, we describe the participants’ characteristics based on three groups: (i) Unlikely to be vaccinated (1.9%), (ii) Unsure about vaccination (16.0%), and (iii) Likely to be vaccinated (82.1%). Fig 1 plots the demographic characteristics of respondents in the respective intention categories, while Table 1 reports the corresponding summary statistics of the HBM, TRA, trust, vaccine preference, and vaccine passport variables. Table 1 also reports the Cronbach’s alpha for the HBM, TRA, and trust variables. The values are all above 0.7, indicating acceptable internal consistency.

**Table 1:** Descriptive statistics of all variables in the national sample (N = 804).

Characteristic	Unlikely to be vaccinated	Unsure about vaccination	Likely to be vaccinated	Cronbach’s alpha
	**(n = 15; 1.86%)**	**(n = 129; 16.04%)**	**(n = 660; 82.09%)**	
	**(M, SD)**	**(M, SD)**	**(M, SD)**	
**Perceived Severity**	2.82 (1.11)	3.69 (0.75)	3.92 (0.70)	0.772
**Perceived Susceptibility**	2.48 (0.97)	3.33 (0.75)	3.89 (0.71)	0.802
**Perceived Barriers**				
Clinical barrier	4.02 (1.14)	3.31 (0.65)	2.87 (0.81)	0.836
Access barrier	3.20 (0.83)	3.24 (0.81)	3.15 (0.94)	0.715
Registration barrier	2.80 (0.87)	3.02 (0.91)	2.41 (1.10)	0.861
Religion barrier	3.03 (1.48)	2.31(0.96)	1.52 (0.84)	0.826
**Perceived Benefits**				
Individual benefits	2.22 (1.39)	3.42 (0.78)	4.09 (0.75)	0.801
Community benefits	2.49 (1.25)	3.59 (0.72)	4.37 (0.59)	0.776
**Attitude**	1.55 (0.70)	3.10 (0.74)	4.39 (0.80)	0.904
**Subjective norms**	1.88 (0.81)	3.45 (0.65)	4.51 (0.57)	0.943
**Trust**				
Vaccine	1.82 (1.12)	3.25 (0.78)	3.99 (0.76)	0.912
Information	2.40 (0.85)	3.37 (0.75)	3.82 (0.65)	0.732
	**n (%)**	**n (%)**	**n (%)**	
**Vaccine Preference**				N.A.
AstraZeneca	0 (0%)	18 (14%)	64 (9.7%)	
Moderna	0 (0%)	8 (6.2%)	26 (3.9%)	
Pfizer	6 (40%)	52 (40%)	346 (52%)	
Sinovac	2 (13%)	23 (18%)	90 (14%)	
Any of the above	7 (47%)	28 (22%)	134 (20%)	
**Vaccine Passport**				N.A.
No/Unsure	14 (93%)	63 (49%)	142 (22%)	
Yes	1 (6.7%)	66 (51%)	518 (78%)	

In Fig 1, the Unsure group’s average age was the smallest (mean = 32 years). There were more females (54%) than males in the Likely group, and participants with tertiary education constitute the largest sample proportion in all three groups. In Table 1, the Likely group shows the largest average scores for perceived severity, perceived susceptibility, perceived benefits, attitude, subjective norms, and trust. It also recorded the smallest average for perceived barriers. The preferred vaccine of choice was Pfizer for all three groups. Only 6.7% of respondents in the Unlikely group were more likely to get vaccinated if vaccine passports were issued for international travel. The percentage is significantly higher at 51% for those unsure about vaccination.

The respondents were dichotomized as likely or unlikely/unsure in the binary logistic regression model. Very few respondents answered that they were unlikely to get vaccinated and hence were merged with those unsure of getting vaccinated. The odds ratio (OR) and the 95% confidence interval are provided in Table 2 [34]. A *p*-value of less than 0.05 was considered statistically significant. As shown in Table 2, the results of the logistic regression identified the factors that predicted the level of acceptance.

**Table 2:** Logistic regression model of COVID-19 vaccine intention.

	Logistic		
	Likely		
	(Ref: Unlikely & Unsure)		
Characteristic	OR^a^	95% CI^b^	*p*-value
Age	1.03	1.00, 1.06	0.032
**Gender**			
Female	—	—	
Male	0.83	0.47, 1.46	0.5
**Education**			
Secondary or lower	—	—	
Diploma	1.37	0.60, 3.14	0.5
Tertiary	1.12	0.52, 2.40	0.8
**Income**			
Less than RM1,000	—	—	
RM1,000–RM3,999	0.49	0.20, 1.18	0.12
RM4,000-RM6,999	0.61	0.24, 1.48	0.3
RM7,000-RM9,999	0.62	0.19, 2.01	0.4
Above RM10,000	0.76	0.17, 3.84	0.7
Severity	1.01	0.64, 1.58	>0.9
Susceptibility	2.14	1.38, 3.38	<0.001
Clinical barriers	1.08	0.66, 1.75	0.8
Access barriers	1.38	0.92, 2.09	0.13
Registration barriers	0.91	0.66, 1.25	0.6
Religion barriers	0.58	0.41, 0.80	0.001
Individual benefits	1.02	0.59, 1.71	>0.9
Community benefits	1.38	0.78, 2.48	0.3
Attitude	2.46	1.69, 3.65	<0.001
Subjective norms	3.79	2.33, 6.35	<0.001
Trust in vaccine	1.78	1.13, 2.81	0.013
Trust in information	0.81	0.48, 1.36	0.4
**Vaccine Preference**			
AstraZeneca	—	—	
Moderna	1.55	0.39, 6.49	0.5
Pfizer	1.11	0.44, 2.76	0.8
Sinovac	0.76	0.26, 2.15	0.6
Any of the above	0.96	0.34, 2.62	>0.9
**Vaccine Passport**			
No/Unsure	—	—	
Yes	1.31	0.72, 2.36	0.4

^a^ OR = Odds Ratio.

^b^ CI = Confidence Interval.

The factors associated with respondents’ vaccination intention are age, susceptibility, religious barriers, attitude, subjective norms, and trust in the vaccine. Age is positively associated with vaccination intention (OR = 1.03, 95% CI [1.0,1.06]; *p* = 0.032). This result implies that older people are more inclined to get vaccinated. The higher the level of perceived susceptibility, the higher the odds of being vaccinated (OR = 2.14; 95% CI [1.38, 3.38]; *p* < 0.001), on controlling for all other predictor variables included in the model. Thus, people who perceive themselves as more prone to COVID-19 infection are more willing to be vaccinated.

Conversely, concerning religious barriers, respondents who reported a higher perception of religious barriers had lower odds of being vaccinated (OR = 0.58; 95% CI [0.41,0.80]; *p* = 0.001), on controlling for all other variables. In other words, individuals who perceive that their religious beliefs or practices are against vaccination are more likely to exhibit uncertainty toward getting vaccinated.

In the context of attitude, a more positive attitude toward vaccination was associated with higher odds of being vaccinated (OR = 2.46; 95% CI [1.69, 3.65]; *p* < 0.001), controlling for all other variables. This means that people with a positive attitude are more likely to take up the vaccine themselves.

Increased perception of subjective norms was associated with higher odds of being vaccinated (OR = 3.79, 95% CI [2.33, 6.35], *p* < 0.001), controlling for all other variables. Hence, people with higher perceived social pressure to get vaccinated are shown to have higher acceptability of the vaccines.

The findings on trust in the vaccine process show that respondents who had a greater level of trust in the vaccine process had higher odds of being vaccinated (OR = 1.78, 95% CI [1.13, 2.81], *p* < 0.013), controlling for all other variables. This result signifies that a high level of trust in the vaccine process is required to reduce public uncertainty toward the vaccines, and thus increase the vaccine uptake.

## Discussion

It is widely recognized that vaccination effectively reduces the risk of getting and spreading the COVID-19 virus [35,36]. However, the success of the vaccination program depends on the willingness of all population segments to be vaccinated, given that significant portions of the population still fall into two segments—those who are unsure about vaccination and those unlikely to get vaccinated. Hence, monitoring vaccine hesitancy is critical, and vaccine programs should carefully consider various incentives to motivate people to get vaccinated, and in particular, these programs should target both population segments. Hence, to optimize COVID-19 vaccine uptake in Malaysia, we conducted this study in order to understand the factors underpinning vaccine hesitancy and to use that information to tailor programs and initiatives that effectively target both these population segments. The findings from this study revealed that the predictors of COVID-19 vaccine hesitancy that are significant at the 5% level of significance are age, susceptibility, religious beliefs, attitude, subjective norms, and trust in the vaccine.

The results showed that the older age groups who perceive themselves to be more prone to COVID-19 infection are more inclined to get vaccinated. This result is in contrast to that of a global survey of 17 countries, which reported higher vaccine hesitancy with increasing age [37]. In contrast, our study showed that the younger age groups are less inclined to get vaccinated. This result is consistent with that of another Malaysian study, that of Chan et al. [38], who found that the younger population was more hesitant to be vaccinated for they perceived the risks of COVID-19 as low. However, while the younger generation seems to be less at risk of severe infection from COVID-19 than the older group, they are more likely to be risk-takers by partaking in social activities during the pandemic. This poses a significant risk as the younger group may be a potential ‘vector’ for spreading the disease in the population, putting the older groups and those with comorbidities at risk of being infected and suffering from severe health outcomes, including death owing to the high infectivity rate of the virus.

Hence, proactive measures should focus on the youth who are less at risk for severe infection effects but can potentially be super-spreaders of the virus. Interestingly, early public information on the virus was focused on the elderly and those with comorbidities, as opposed to the youth. More intensive public campaigns and youth organizations can do their part to reach out and connect to the youth to influence their perception of vaccination.

Further, the results also indicate that social influence, a person’s perception that people closest to them (family, friends, and peers) think they should get vaccinated, can help in boosting vaccination intention. The perception that family and friends are pro-vaccination can affect vaccine uptake. The belief that people close to them would want them to get vaccinated resulted in high odds to be vaccinated, similar to that in the US and Ireland [28,39]. With social influence playing a crucial part in boosting vaccination intention, encouraging youths to accept the vaccine could reinforce their intention to be vaccinated.

Respondents who reported a higher perception of religious barriers were more likely to be unsure/unlikely about their vaccination intention. This result suggests that religion can be a barrier to vaccination programs. Other Malaysian studies have also reported how religion impedes people’s intention to take the COVID-19 vaccine [10,19,40]. Thus, it is important to involve religious and community leaders to promote vaccination acceptance. While the Ministry of Health’s vaccination efforts and programs continue at the national level, public health authorities should seek partnerships with faith-based organizations to address religious objections to vaccination, particularly a wrong perception or lack of community trust, through open dialogues. In this study, the questions on religious barriers include “My religion prohibits me from getting vaccinated” and “I believe that the COVID-19 vaccine contains ingredients prohibited by my religion.” The responses suggest that these issues are a concern among the respondents. It is important that public health authorities and medical experts promptly respond and clarify these concerns. Adopting this approach will allow direct contact between medical experts, religious authorities, and individuals concerned about these issues. This type of intervention to understand, inform, and influence different target audiences is essential in increasing the intention to be vaccinated. Therefore, promoting uptake through targeted population outreach is one way to mitigate vaccine hesitancy among religious communities. These efforts include municipality level vaccination outreach programs and events tailored to religious communities.

In addition, Ministry of Health personnel, including medical officers in hospitals that have strong links with religious communities, should be recruited for promotional campaigns and outreach programs with their patients to counter vaccine hesitancy. This type of intervention of partnering with religious leaders and receiving their endorsement can contribute to vaccine uptake, as evidenced among the Ultra-Orthodox in Israel [41]. Further, communal leaders in Israel were also recruited to encourage compliance throughout the vaccination campaign.

Next, people with a more positive attitude are more likely to take up the vaccine themselves, augmenting the finding in the Malaysian context by Wong et al. [42], who reported that older participants with positive attitudes to vaccination were 16 times more likely to be vaccinated. This phenomenon is consistent with the findings of studies conducted in the United Kingdom [43] and the United States [28]. Since attitude is a significant driver of vaccine hesitancy, there is a need to shape attitude using various modalities, such as peer groups and local champions apart from general campaigns. A possible solution is for policymakers to enhance public education awareness programs and campaigns, communication, and messaging to provide a continuous flow of information in order to dispel fears. This includes emphasizing the importance of individual benefits of vaccination in safeguarding them from the virus, which in turn influences the community. This information and visuals can reassure individuals, as an Israeli study [41, p.10] highlighted: “If so many Israelis are queueing up to get vaccinated voluntarily, then this is probably a good thing for me as well.” This approach should include prompt coordination by senior public health officials from the Ministry of Health, experts, and public figures to respond to anti-vax messages using mass media, social media, and other channels.

Last, a high level of trust in the vaccine, that is, trust in the institutions and regulatory agencies, is vital to reduce public uncertainty toward vaccines, which would therefore increase the vaccine’s uptake. Vaccine hesitancy is attributed mainly to the lack of trust in vaccines’ efficacy and safety issues. This finding has been echoed by prior studies as well [19,38]. This is particularly important for those uncertain about vaccine efficacy and safety, who would be put off easily if their attempts to fix an appointment in the first instance failed. Hence, to improve vaccine uptake, initiatives and strategies to bring the vaccines to the people using mobile or pop-up vaccine clinics should be encouraged and may be the solution to mitigate the risk of the people not being vaccinated. These initiatives include having medical officers available to address any queries from people about the impact and efficacy of the vaccines. Easy access to vaccine facilities and information about the vaccine will go a long way to maintain positive public views toward vaccine uptake.

## Conclusion

This study examined factors contributing to vaccine hesitancy from a developing country’s perspective. It revealed that the predictors that significantly affected COVID-19 vaccine hesitancy in Malaysia are age, susceptibility, religious beliefs, attitude, subjective norms, and trust in the vaccine. Although some of these factors are not unique to Malaysia, the findings of this study can provide a better understanding of the reasons for vaccine hesitancy within the context of Malaysia. Hence, the country’s public health authorities and other relevant stakeholders can formulate effective strategies, initiatives, and outreach programs to facilitate immunization programs in Malaysia against future health pandemics. Lessons from the Malaysian study may provide valuable insights for other developing countries in addressing issues related to vaccine hesitancy.

Nevertheless, the study has several limitations. First, the data collection using a self-administered questionnaire was conducted via an online portal. Hence, the respondents were skewed toward the urban population with broader access to the internet and higher literacy levels. Generalization of the findings to the overall population and assumptions about the urban residents could cause policymakers and relevant authorities to put in place strategies that consider this segment of the population more than the rural population. Future studies should cover nationally representative households, which was impossible during the pandemic owing to the restrictions imposed during the lockdowns. Second, this study used cross-sectional data where individuals’ perception of their vaccination intention was measured at a single point in time. Perception, however, may change over time. Regarding future work, a longitudinal study would provide further insights into the effectiveness of any intervention strategies.

## Supporting information

S1 Appendix Questionnaire. (DOCX)
