# Supplementary material for: Key predictors of COVID-19 vaccine hesitancy in Malaysia: An integrated framework
Source: PLoS One. 2022 May 23;17(5):e0268926. doi: 10.1371/journal.pone.0268926 (PMC9126375; doi:10.1371/journal.pone.0268926)
Supplement: S1 Appendix — (DOCX) [file pone.0268926.s001.docx]

**S1 Questionnaire.**

**SECTION A**

**Perceived Severity**

In this section, we would like to know your personal beliefs regarding the negative effects of contracting Covid-19.

For each of the following list of statements, please pick the option which best represents your opinion.

|  |  | Strongly agree | Somewhat agree | Neither agree nor disagree | Somewhat disagree | Strongly disagree |
| --- | --- | --- | --- | --- | --- | --- |
| 1 | If I get Covid-19, I will lose income | 5 | 4 | 3 | 2 | 1 |
| 2 | If I get Covid-19, I will die | 5 | 4 | 3 | 2 | 1 |
| 3 | If I get Covid-19, other members in my home will get sick | 5 | 4 | 3 | 2 | 1 |
| 4 | A person who contracts Covid-19 will die if not treated | 5 | 4 | 3 | 2 | 1 |
| 5 | I believe that Covid- 19 has serious negative consequences | 5 | 4 | 3 | 2 | 1 |
| 6 | I believe that Covid- 19 is a severe health problem | 5 | 4 | 3 | 2 | 1 |

**Perceived Susceptibility**

In this section, we would like to know your personal beliefs regarding vulnerability to infection with Covid-19.

For each of the following list of statements, please pick the option which best represents your opinion.

|  |  | Strongly agree | Somewhat agree | Neither agree nor disagree | Somewhat disagree | Strongly disagree |
| --- | --- | --- | --- | --- | --- | --- |
| 1 | Covid-19 can happen to many people, including my family, loved ones and friends | 5 | 4 | 3 | 2 | 1 |
| 2 | Covid-19 can happen anytime to anyone, such as a healthy individual | 5 | 4 | 3 | 2 | 1 |
| 3 | I am at risk of getting Covid-19 | 5 | 4 | 3 | 2 | 1 |
| 4 | The chance of me contracting Covid-19 is high | 5 | 4 | 3 | 2 | 1 |
| 5 | It is likely that I will get Covid-19 | 5 | 4 | 3 | 2 | 1 |

**Clinical Barriers**

In this section, we would like to know your perceived barriers to Covid-19 vaccination, defined as the belief that being vaccinated is restricted due to various factors.

For each of the following list of statements, please pick the option which best represents your opinion.

|  |  | Strongly agree | Somewhat agree | Neither agree nor disagree | Somewhat disagree | Strongly disagree |
| --- | --- | --- | --- | --- | --- | --- |
| 1 | Taking the Covid-19 vaccine will result in certain side effects to my body | 5 | 4 | 3 | 2 | 1 |
| 2 | My body may react adversely to the Covid-19 vaccine | 5 | 4 | 3 | 2 | 1 |
| 3 | I will get sick from the Covid-19 vaccine | 5 | 4 | 3 | 2 | 1 |
| 4 | I will die from the Covid-19 vaccine | 5 | 4 | 3 | 2 | 1 |
| 5 | I am concerned about whether Covid-19 vaccines are safe | 5 | 4 | 3 | 2 | 1 |
| 6 | I feel that not enough research has been done on Covid-19 vaccines | 5 | 4 | 3 | 2 | 1 |

**Access/Logistics Barriers**

For each of the following list of statements, please pick the option which best represents your opinion.

|  |  | Strongly agree | Somewhat agree | Neither agree nor disagree | Somewhat disagree | Strongly disagree |
| --- | --- | --- | --- | --- | --- | --- |
| 1 | It is inconvenient to get the Covid-19 vaccine as I have no control over the time and place of vaccination | 5 | 4 | 3 | 2 | 1 |
| 2 | There is a shortage of the Covid-19 vaccine | 5 | 4 | 3 | 2 | 1 |
| 3 | The Covid-19 vaccine is not easily accessible to me | 5 | 4 | 3 | 2 | 1 |
| 4 | The Covid-19 vaccine is costly for me | 5 | 4 | 3 | 2 | 1 |

**Registration Barriers**

For each of the following list of statements, please pick the option which best represents your opinion.

|  |  | Strongly agree | Somewhat agree | Neither agree nor disagree | Somewhat disagree | Strongly disagree |
| --- | --- | --- | --- | --- | --- | --- |
| 1 | It is inconvenient for me to register for Covid-19 vaccination via the 'MySejahtera' mobile application | 5 | 4 | 3 | 2 | 1 |
| 2 | It is inconvenient for me to register for Covid-19 vaccination via the website | 5 | 4 | 3 | 2 | 1 |
| 3 | It is inconvenient for me to register for Covid-19 vaccination via phone call | 5 | 4 | 3 | 2 | 1 |
| 4 | Overall, it is inconvenient for me to register for Covid-19 vaccination | 5 | 4 | 3 | 2 | 1 |

**Religious Barriers**

For each of the following list of statements, please pick the option which best represents your opinion.

|  |  | Strongly agree | Somewhat agree | Neither agree nor disagree | Somewhat disagree | Strongly disagree |
| --- | --- | --- | --- | --- | --- | --- |
| 1 | My religion prohibits me from getting vaccinated | 5 | 4 | 3 | 2 | 1 |
| 2 | I believe that the Covid-19 vaccine contains ingredients prohibited by my religion. | 5 | 4 | 3 | 2 | 1 |

**Perceived Benefits**

In this section, we would like to know your personal beliefs regarding the positive effects of being vaccinated against Covid-19.

For each of the following list of statements, please pick the option which best represents your opinion.

|  |  | Strongly agree | Somewhat agree | Neither agree nor disagree | Somewhat disagree | Strongly disagree |
| --- | --- | --- | --- | --- | --- | --- |
| 1 | Covid-19 vaccines will work in preventing the disease | 5 | 4 | 3 | 2 | 1 |
| 2 | Covid-19 vaccines will be effective in preventing Covid-19 | 5 | 4 | 3 | 2 | 1 |
| 3 | If I get the vaccine, I will be less likely to get Covid-19 | 5 | 4 | 3 | 2 | 1 |
| 4 | Having myself vaccinated against Covid-19 is beneficial for the health of others in my community | 5 | 4 | 3 | 2 | 1 |
| 5 | Covid-19 vaccines protect the health of my community | 5 | 4 | 3 | 2 | 1 |
| 6 | Covid-19 vaccines will enable free travel within and outside of the country | 5 | 4 | 3 | 2 | 1 |

**Intention**

Please indicate the **likelihood** that you will:

|  |  | Very likely | Somewhat likely | Neither likely nor unlikely | Somewhat unlikely | Very unlikely |
| --- | --- | --- | --- | --- | --- | --- |
| 1 | Consider getting vaccinated against Covid-19 | 5 | 4 | 3 | 2 | 1 |
| 2 | Try to get vaccinated against Covid-19 | 5 | 4 | 3 | 2 | 1 |
| 3 | Actually get vaccinated for Covid-19 | 5 | 4 | 3 | 2 | 1 |
| 4 | Get vaccinated if a physician offered you Covid-19 vaccines | 5 | 4 | 3 | 2 | 1 |

**Attitude**

Please indicate how much you feel that getting vaccinated for Covid-19 is: "Unfavourable" (1) to "Favourable" (5)

| 1 | 2 | 3 | 4 | 5 |
| --- | --- | --- | --- | --- |

Please indicate how much you feel that getting vaccinated for Covid-19 is: "Bad" (1) to "Good" (5)

| 1 | 2 | 3 | 4 | 5 |
| --- | --- | --- | --- | --- |

Please indicate how much you feel that getting vaccinated for Covid-19 is: "Harmful" (1) to "Beneficial" (5)

| 1 | 2 | 3 | 4 | 5 |
| --- | --- | --- | --- | --- |

Please indicate how much you feel that getting vaccinated for Covid-19 is: "Foolish" (1) to "Wise" (5)

| 1 | 2 | 3 | 4 | 5 |
| --- | --- | --- | --- | --- |

**Subjective Norms**

For each of the following list of statements, please pick the option which best represents your opinion.

|  |  | Strongly agree | Somewhat agree | Neither agree nor disagree | Somewhat disagree | Strongly disagree |
| --- | --- | --- | --- | --- | --- | --- |
| 1 | My family members think I should get the Covid-19 vaccine | 5 | 4 | 3 | 2 | 1 |
| 2 | My close friends think I should get the Covid-19 vaccine | 5 | 4 | 3 | 2 | 1 |
| 3 | People who are like me will get vaccinated for Covid-19 | 5 | 4 | 3 | 2 | 1 |
| 4 | People who are important to me will get vaccinated for Covid-19 | 5 | 4 | 3 | 2 | 1 |
| 5 | People who are important to me think that I should get the Covid-19 vaccine | 5 | 4 | 3 | 2 | 1 |
| 6 | People who influence my behaviour think that I should get vaccinated against Covid-19 | 5 | 4 | 3 | 2 | 1 |

**Trust**

For each of the following list of statements, please pick the option which best represents your opinion.

(Note: The National Pharmaceutical Regulatory Agency (NPRA) is responsible for approving Covid-19 vaccines administered in Malaysia.)

|  |  | Strongly trust | Somewhat trust | Neither trust nor distrust | Somwhat distrust | Strongly distrust |
| --- | --- | --- | --- | --- | --- | --- |
| 1 | When it comes to the Covid-19 vaccine process, how much do you trust the World Health Organization? | 5 | 4 | 3 | 2 | 1 |
| 2 | When it comes to the Covid-19 vaccine process, how much do you trust the National Pharmaceutical Regulatory Agency (NPRA)? | 5 | 4 | 3 | 2 | 1 |
| 3 | Overall, how much do you trust that the organizations involved in the Covid-19 vaccine make their decisions with the public's best interest in mind? | 5 | 4 | 3 | 2 | 1 |
| 4 | Overall, how much do you trust that all the involved organizations do a good job when it comes to the Covid- 19 vaccine? | 5 | 4 | 3 | 2 | 1 |

How much do you trust or distrust each of the following as a source of information about the Covid-19 pandemic?

|  |  | Strongly trust | Somewhat trust | Neither trust nor distrust | Somwhat distrust | Strongly distrust |
| --- | --- | --- | --- | --- | --- | --- |
| 1 | Mainstream media and news such as TV news, newspapers, radio news | 5 | 4 | 3 | 2 | 1 |
| 2 | Social media such as Instagram, Facebook, TikTok, Twitter, YouTube, etc. | 5 | 4 | 3 | 2 | 1 |
| 3 | Public health officals or agencies | 5 | 4 | 3 | 2 | 1 |

For each of the following list of statements, please pick the option which best represents your opinion.

|  |  | A great deal | A lot | A moderate amount | A little | None at all |
| --- | --- | --- | --- | --- | --- | --- |
| 1 | During a major crisis, the government informs you about the concerned crisis. How much trust do you generally have in information provided by the Malaysian government about Covid-19? | 5 | 4 | 3 | 2 | 1 |
| 2 | How much trust do you have in measures already taken by the Malaysian government against Covid-19? | 5 | 4 | 3 | 2 | 1 |
| 3 | How much trust do you have in the Malaysian government with respect to fighting the Covid-19 pandemic? |  |  |  |  |  |
| 4 | What do you think of the decisiveness of the Malaysian government in taking safety measures against Covid-19 in Malaysia? | 5 | 4 | 3 | 2 | 1 |
| 5 | How much trust do you generally have in the Malaysian government, irrespective of crisis management? | 5 | 4 | 3 | 2 | 1 |

Have you been vaccinated against Covid-19 (includes receiving at least 1 vaccine dosage)?

Yes

No

Have you registered for Covid-19 vaccination?

Yes

No

Currently, people are not able to select which Covid-19 vaccine they receive. Would you prefer to decide which Covid-19 vaccine you receive?

Yes

No

Doesn’t matter

If you had a choice, which Covid-19 vaccine would you **prefer** to take?

AstraZeneca

Moderna

Pfizer

Sinovac

Any of the above

If you had a choice, which Covid-19 vaccine would you **avoid** taking?

AstraZeneca

Moderna

Pfizer

Sinovac

Doesn’t matter

Would you be **more likely** to be vaccinated against Covid-19 if a vaccine passport was subsequently issued to you for international travel?

Yes

No

Unsure

**SECTION B**

**Demographics**

Nationality:

Malaysian Citizen
 Malaysian Permanent Resident

Foreigner

Age (years):

18 - 30

31 - 40

41 - 50

51 - 60

61 - 70

71 - 80

Above 80

Gender:

Male

Female

Ethnicity:

Malay

Chinese

Indian

Bumiputera Sabah

Bumiputera Sarawak

Others (please state):

Religion:

None

Buddhism

Catholicism

Christianity

Islam
 Hinduism
 Taoism
 Others (please specify):

Marital Status:

Never married
 Married
 Divorced/Permanently separated

Widowed

Highest Education Level attained:

None
 Primary Education

Secondary Education

Diploma
 Tertiary Education

Employment Status:

Self-Employed
 Employed full time
 Employed part time
 Unemployed looking for work Unemployed not looking for work

Domestic homemaker Retired
 Student
 Disabled

Average Monthly Income (RM):

Less than RM1,000

RM1,000 - RM3,999

RM4,000 - RM6,999

RM7,000 - RM9,999

Above RM10,000

Please indicate any existing chronic diseases that you may have:
(Note: A chronic illness is a long-term health condition that may not have a cure)

None
 Alzheimer diseases/ Dementia

Arthritis

Cancer

Chronic obstructive pulmonary disease (COPD)

Diabetes

Epilepsy

Heart Disease

Mood disorders (bipolar, cyclothymic, depression)

Parkinson diseases

Stroke
 Others (please specify):

Except for Covid-19 vaccines, have you received any vaccines (e.g. flu shot) in the past 18 months?

Yes

No

Unsure

Please rate your overall health status
 Very poor

Poor

Average

Good

Very good
